# Compensation for wind drift prevails for a shorebird on a long-distance, transoceanic flight

**DOI:** 10.1186/s40462-022-00310-z

**Published:** 2022-03-07

**Authors:** Jennifer A. Linscott, Juan G. Navedo, Sarah J. Clements, Jason P. Loghry, Jorge Ruiz, Bart M. Ballard, Mitch D. Weegman, Nathan R. Senner

**Affiliations:** 1 Department of Biological Sciences, University of South Carolina, 715 Sumter Street, Columbia, SC 29208 USA; 2 Estacion Experimental Quempillén, Facultad de Ciencias, Universidad Austral de Chile, Ancud, Chiloé Chile; 3 Instituto de Ciencias Marinas y Limnológicas, Universidad Austral de Chile, Valdivia, Chile; 4 School of Natural Resources, University of Missouri, 103 Anheuser-Busch Natural Resources Building, Columbia, MO 65211 USA; 5 Texas A&M University, Kingsville, 700 University Blvd., MSC 218, Kingsville, TX 78363 USA; 6 Department of Biology, University of Saskatchewan, 112 Science Place, Saskatoon, SK S7N 5E2 Canada

**Keywords:** Migration, Flight, Wind conditions, Avian migratory strategy, Ecological barrier

## Abstract

**Background:**

Conditions encountered *en route* can dramatically impact the energy that migratory species spend on movement. Migratory birds often manage energetic costs by adjusting their behavior in relation to wind conditions as they fly. Wind-influenced behaviors can offer insight into the relative importance of risk and resistance during migration, but to date, they have only been studied in a limited subset of avian species and flight types. We add to this understanding by examining in-flight behaviors over a days-long, barrier-crossing flight in a migratory shorebird.

**Methods:**

Using satellite tracking devices, we followed 25 Hudsonian godwits (*Limosa haemastica*) from 2019–2021 as they migrated northward across a largely transoceanic landscape extending > 7000 km from Chiloé Island, Chile to the northern coast of the Gulf of Mexico. We identified in-flight behaviors during this crossing by comparing directions of critical movement vectors and used mixed models to test whether the resulting patterns supported three classical predictions about wind and migration.

**Results:**

Contrary to our predictions, compensation did not increase linearly with distance traveled, was not constrained during flight over open ocean, and did not influence where an individual ultimately crossed over the northern coast of the Gulf of Mexico at the end of this flight. Instead, we found a strong preference for full compensation throughout godwit flight paths.

**Conclusions:**

Our results indicate that compensation is crucial to godwits, emphasizing the role of risk in shaping migratory behavior and raising questions about the consequences of changing wind regimes for other barrier-crossing aerial migrants.

**Supplementary Information:**

The online version contains supplementary material available at 10.1186/s40462-022-00310-z.

## Background

The energetic cost of movement can vary substantially during migration. Far-ranging migratory animals often minimize this cost by selecting routes where topography or predictable flow regimes offer lower resistance to forward progress [1, 2], effectively extending their distance traveled per unit of energetic power. In many cases, however, their routes also pass through regions where elevated energetic costs are unavoidable, including high-resistance landscapes that bisect the migratory corridor and high-risk landscapes (often termed “barriers”) that offer no opportunities to rest or refuel if energy stores run low [3]. Evaluating behaviors in these regions can therefore offer insights into optimal strategies, the evolution of migratory routes, and the ways that migrants may need to respond to future change [4].

For birds, the cost of migratory movement is largely shaped by the wind field aloft—a landscape that is spatially complex, temporally dynamic, and often unpredictable, particularly if local conditions at the departure site are only weakly correlated with conditions further along a route [5]. When wind flow opposes their preferred range of movement, birds can adjust their heading and airspeed to fully (or ‘completely’) compensate for lateral displacement [6]. Partially compensating, drifting, and overcompensating [7] have also been documented along flight paths, suggesting that birds rely on multiple behavioral tactics to minimize total energetic costs and reduce the risk of depleting energy stores during vulnerable phases of the migratory journey [8].

Several theoretical predictions outline how birds may integrate these tactics into a movement strategy. When traveling through changing wind fields, the energetically optimal strategy is predicted to be drifting early and increasing compensation as the destination approaches [6, 9]. The *en masse* springtime movements of birds in the Northern Hemisphere appear to follow this pattern [10, 11], as do the movements of many Arctic-breeding waders [9]. Species- and population-specific studies, however, have also found deviations from this pattern, suggesting that behavioral strategies can be route-dependent. Generally unfavorable winds at a departure site, for instance, may drive lower rates of wind selectivity and higher rates of partial compensation [12], while variations in risk due to global wind regimes or geographic barriers may facilitate more flexible behavioral changes throughout the migratory journey [13, 14]. In other cases, behavioral strategies may also be influenced by body size and season [11], or potentially by stopover site use. In fact, most empirical examinations of drift and compensation—as well as the theoretical predictions on which they are based—have been performed in the context of frequently stopping migrants, such as fly-and-forage raptors and nocturnally migrating passerines. For migrants that make lengthy nonstop flights, presumably along stable, wind-optimized migratory corridors [2], optimal strategies are less clear.

Additionally, behaviors may also be subject to non-adaptive limitations. Full compensation has been hypothesized to require visual cues, which enable individuals to gauge their rate of displacement from the preferred range by watching the angular displacement of approaching landmarks around their body axes [15] and adjusting their airspeed or heading to compensate accordingly [16]. Relatively featureless landscapes, such as open oceans or pack ice [6, 17], or landscapes overflown at high altitudes that surpass thresholds of visual detection [18, 19] may then constrain compensation in locations where it would be otherwise optimal [6], particularly if they are overflown at night [20]. Radar studies have found reductions in drift tolerance along visually salient landmarks like rivers [21] and coastlines [22, 23], though the lack of radar coverage over uninhabited areas—such as the open ocean—means that the generality of these conclusions is potentially limited. Individual tracking studies, by contrast, have revealed remarkably direct tracks over open ocean [24], albeit for a fraction of the time and distance that other migratory birds spend aloft. A more complete picture of the strategies and limitations that shape in-flight behavior will thus require empirical testing across a broader diversity of avian migrations.

Here, we offer an examination for one such flight: the marathon, transoceanic flight of an Arctic-breeding shorebird, the Hudsonian godwit (*Limosa haemastica*; hereafter, godwits). Godwits spend the austral summer in coastal Chile and Argentina and migrate northward through the midcontinental United States to breed in arctic and subarctic Alaska and Canada. We focus on the initial stage of this journey, which comprises one of the longest recorded nonstop flights of any landbird species and takes place largely over the open Pacific Ocean and Gulf of Mexico [25]. Godwits making this flight have few or no opportunities to stop, and they traverse several global wind regimes that differ in directionality and strength along the way, including the South Pacific Subtropical Anticyclone (SPSA), the trade winds, and the Intertropical Convergence Zone (ITCZ). To this journey we apply two theoretical predictions outlined above: that godwits will (1) preferentially drift after departure and begin to increase compensation as they approach North America, and (2) exhibit particularly high rates of drift over open ocean. Given these predictions, as well as the extensive distance and time spent aloft, we additionally predict that (3) winds experienced during this flight will influence where individuals cross into North America along the Northern Gulf of Mexico. To test these predictions, we developed a circular framework for behavioral classification that allows movement toward a flexible stopover region rather than a single, fixed destination. This framework leverages individual tracking data into a broader illumination of the behaviors necessary to safely navigate a long-distance, barrier-crossing migratory flight.

## Methods

### Transmitter attachment

To monitor godwit movements during northward migration, we captured godwits during the non-breeding season on the Chiloé Archipelago, Chile (41°49′S–73°37′W and 42°28′S-73°41′W) prior to migratory departure (see details in [26]) and affixed solar-powered satellite transmitters to 54 adults. We chose both males and females of larger body sizes to control for the effects of transmitter weight; in all cases, the combined weight of transmitters and harnesses comprised < 3% of body mass at capture. In total, we attached 29 Argos Solar Platform Terminal Transmitters (PTTs) weighing 5 g (Microwave Technologies Ltd.) and 25 Pinpoint GPS/Argos Solar S Transmitters weighing 6.6 g (Lotek) using leg-loop harnesses made of nylon or silicon cord.

Both the frequency and precision of location estimates varied between transmitter models. The 5-g devices transmitted Argos locations according to a pre-programmed duty cycle (5-h transmitting/24-h charging); the 6.6-g devices transmitted GPS locations every 2 h and Argos locations opportunistically. To reduce spatial error, we retained standard-quality Argos locations (classes 3, 2, and 1) and passed those of lower-quality (classes 0, A, and B) through a Douglas-Kalman hybrid filter with a maximum realistic rate of movement of 130 km h^−1^ [27] and a maximum redundant distance of 5 km [28]. For GPS locations with a ± 10-m resolution, we applied a rate-based filtering algorithm that removed locations requiring similarly implausible flight speeds (> 130 km h^−1^).

### Preparing flight tracks

**Fig. 1:**
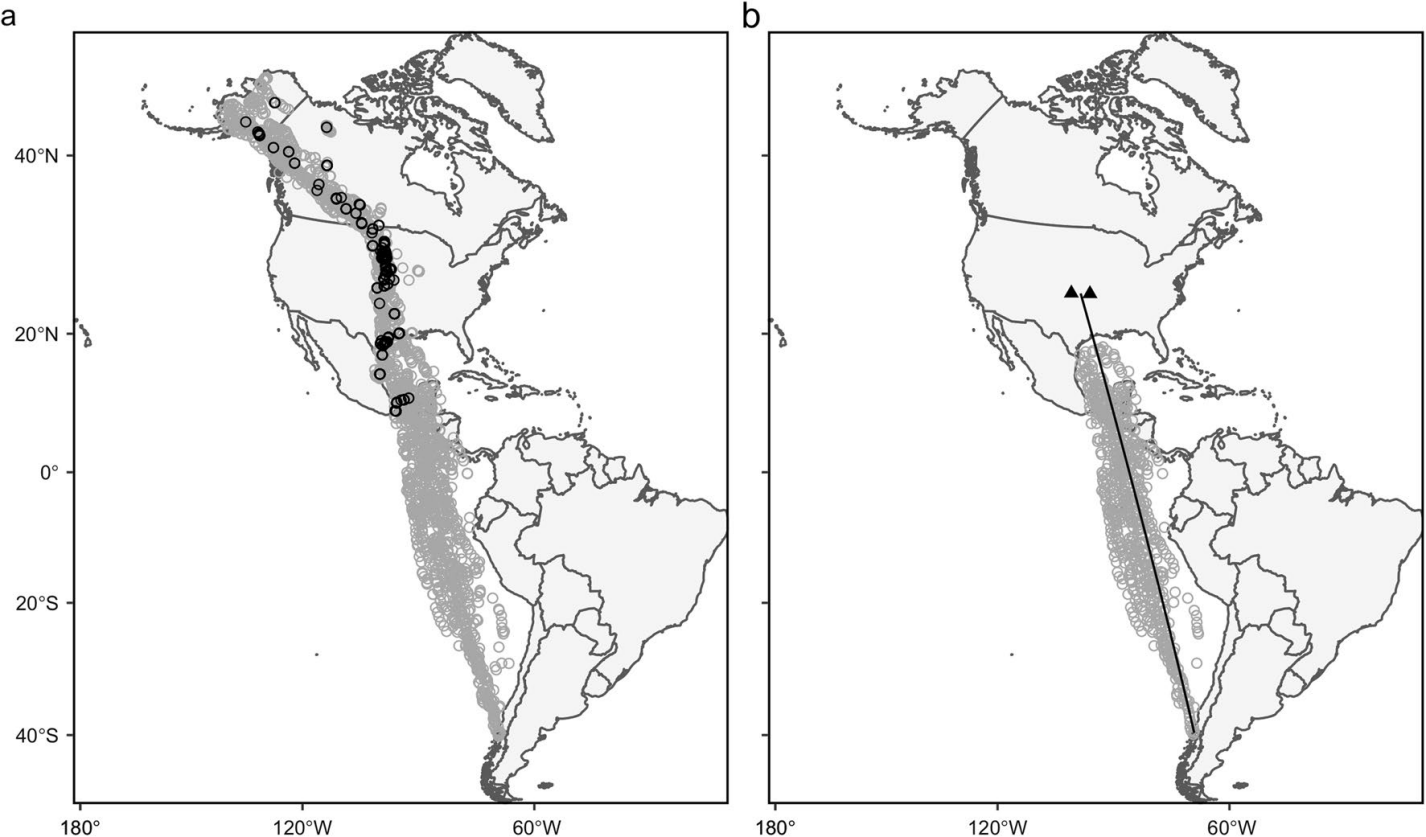
Godwit northward migratory flight, 2019–2021. **a** All locations reported by tracking devices from departure (Chiloé Island, Chile) to breeding sites (Alaska). Gray circles are points during flight; black circles are points during stopover (groundspeed < 3 ms^−1^ and distance traveled < 15 km). **b** Locations included in our behavioral analyses. Triangles denote the westernmost and easternmost boundaries of the main stopover region, toward which godwits are traveling. Black line depicts the shortest distance (i.e., Great Circle) route from departure to the midpoint of these boundaries

Godwits from Chiloé mainly breed in Alaska. To reach their breeding sites, godwits make a series of flights beginning with a transhemispheric non-stop flight that proceeds over the Pacific Ocean, Central America, and the Gulf of Mexico along a south/north axis. After crossing the Gulf of Mexico, the remainder of their migratory journey occurs over land (Fig. 1; [29]). We focused on godwit behavior during the initial flight stage—which spans from Chiloé to the northern Gulf Coast (hereafter, ‘NGC’) and is generally flown non-stop [25]—for several reasons. First, flights of this nature are seldom featured in behavioral studies (but see [30]), particularly at this level of precision. Second, this flight also improves interpretability of our analyses, as both vertical and lateral movements here are believed to be influenced primarily by wind conditions aloft. By contrast, overland flights after godwits cross into North America are more complex, often involving lengthy searches for spatially dispersed wetland stopover habitat. Behavior over land thus may be influenced by a combination of wind conditions and underlying habitat suitability, which we do not consider here.

To establish godwits’ intended migratory direction, we used their convergence on a main stopover region in midcontinental North America. The region is relatively narrow—encompassing the eastern portions of Kansas, Nebraska, South Dakota, and North Dakota—and contains a high concentration of the ephemeral seasonal wetlands that godwits use to refuel. While godwits do not revisit particular stopover sites, their convergence on and passage through this region is nonetheless highly predictable [25]. We adapted methods from Pearse et al. [31] to identify where godwit tracks exhibited the least latitudinal dispersion in the approach to and while crossing this main stopover region (see Additional file 1 for details). The western and eastern boundaries of this area, as well as their geographic midpoint, served as approximations of the godwits’ overall preferred direction. Dispersion distances and midpoints were calculated on an ellipsoid using functions from the *geosphere* package [32].

We selected only movements within godwit flight tracks that were associated with their initial flight. From the first offshore location indicative of directional movement away from Chiloé, we used current ground speed and straight-line flight distance from the last onshore location to estimate an hourly time window during which an individual likely departed. All locations prior to this time were removed. Next, tracks were truncated at the first overland location after crossing the Gulf of Mexico, where many individuals began searching for stopover sites. In between these endpoints, we calculated distance, ground speed, turning angle, and heading from each location to the next consecutive location using functions from the R package *move* [33]. Overland locations where ground speeds fell below 3 ms^−1^ [34] and consecutive movements traversed < 15 km were indicative of either stopover or transmitter failure and removed from flight tracks. The result was a northward track for each individual, comprising either Argos or Argos and GPS locations, and reflecting directional migratory flight from Chiloé to the NGC [35].

Finally, we performed two additional data thinning steps in preparation for behavioral analyses. To reduce the influence of error and spatial dependence in closely spaced locations, we thinned tracks to a maximum of one location per hour (i.e., the temporal resolution of our wind data), removing locations with lower quality or, where quality was equivalent, locations at random. To reduce the likelihood of dramatic shifts in wind conditions between very widely separated locations, we also removed locations preceding a reporting gap > 12 h. This excluded locations immediately preceding the regular 24-h “off” period in 5-g transmitters and locations preceding consecutive incidental gaps in 6.6-g transmitters, which became especially pronounced in one transmitter deployed continuously for several years. The resulting tracks (*n* = 28, with *n* = 689 locations total) contained, on average, one location every 3.25 ± 0.73 h for the 5-g devices and one every 3.15 ± 0.41 h for the 6.6-g devices (Fig. 1).

### Wind conditions during flight

We linked each location with the range of possible wind conditions that godwits were likely experiencing at the time. Conditions were retrieved from the European Center for Medium-Range Weather Forecasts ERA5 climate reanalysis dataset, retrieved from the Copernicus Climate Data Store [36]. For each godwit location, we extracted the east/west (*u*) and north/south (*v*) components of the wind vector at the nearest hour via bilinear interpolation of the nearest points, and for seven altitudinal pressure levels within the known range of avian flight heights: 1000, 925, 850, 775, 700, 600, and 500 hPa, corresponding to altitudes of roughly 100, 750, 1500, 2250, 3000, 3400 and 5500 m above sea level (m.a.s.l.), respectively. We then calculated the total magnitude (ms^−1^) and mathematical direction of the wind flow (i.e., the direction the wind is flowing *towards*) for each location at each altitude using vector trigonometry.

The exact wind conditions that godwits experienced at a location depended on their flight altitude. Though we lacked direct information on altitude, migrating birds are known to alter their flight height dynamically in response to atmospheric conditions [23, 37]. In particular, Black-tailed godwits (*Limosa limosa*) migrating from Europe to Africa change altitude in order to exploit optimal wind conditions and reduce the thermoregulatory burden of powered flight over the Sahara [27]. Since our godwits likely did not experience similarly extreme temperatures during this stage of migration—they travel during the austral fall/boreal spring along a largely transoceanic corridor—we considered wind support alone to be the best predictor of altitude during this period of continuous flight.

To validate this assumption and define the range of possible flight altitudes, we analyzed observed ground speeds in relation to wind conditions aloft. Ground speed remains among the best indicators of the winds birds are experiencing during flight and is commonly used to estimate flight altitude [38]. In total, we built seven linear mixed-effects models (LMMs) to evaluate how ground speed is affected by wind conditions at various altitudes. Three models allowed individuals to fly at a constant altitude (either 100, 750, or 1500 m.a.s.l.). The remaining four models allowed individuals to fly at the altitude offering optimal support, calculated either in the preferred direction of flight (*θ*_*d*_) or the direction of the next location along the realized flight track (*θ*_*t*_), selected from either the lower five altitudes or all seven altitudes. Wind support (ws, ms^−1^) was calculated as:

$$ws = \cos \left( {atan2(u,v) - \frac{\theta \pi }{{180}}} \right) \times \sqrt {u^{2} \times v^{2} }$$

In this equation, $\theta$ refers to the direction of travel, while *u* and *v* refer to the east/west and north/south components of the wind vector, respectively. These models, as well as all LMMs hereafter, initially included year and individual as random intercepts to account for spatial dependence and individual differences [39]. Models were constructed with the R package *lme4* [40] and compared using corrected Aikake’s Information Criterion (AIC_c_) scores [41]. We selected the model with the lowest score or, where competing models demonstrated no substantial difference (ΔAIC_c_ < 2), the simpler of the models.

With this information, we assigned the most likely wind conditions to each godwit location. To evaluate changes in these conditions, we organized the migratory corridor into seven latitudinal bands (~ 10° each) and used circular statistics to evaluate within-band scalar means of wind speed, circular mean directions ($\overline{\theta }$), and the mean resultant length ($\overline{R}$) of flow vectors, where $\overline{R}$ is a statistic between 0 and 1 that indicates the spread of the mean direction (i.e., 0 = large spread; 1 = concentrated at a single value). We confirmed the non-uniformity of wind flow within each band using Rayleigh tests. To assess the robustness of our assigned wind conditions to imprecision in altitude estimation, we used a Pearson’s product-moment correlation for wind direction and wind support at two different altitudes: the altitude offering the maximum wind support and the altitude offering the next-highest wind support. Analyses of circular data were performed with functions from the *circular* package [42].

### Classifying in-flight behavior

To test our behavioral predictions, we assigned one behavior to each godwit location based on the direction of wind flow and the godwit’s subsequent movement. While several methodologies have already been developed for behavioral classifications (e.g., [13, 43]), they generally require a single location that can serve as the migratory destination, such as a fixed breeding or stopover site toward which an individual consistently navigates. In our case, and indeed for many other species that exhibit more flexible movements during the early stages of migration [44], these methodologies may be problematic. Godwits navigate toward a *range* of suitable stopover destinations in midcontinental North American [25]. To preserve this range in our calculations, we developed a new workflow for assigning in-flight behaviors, which utilizes an intuitive circular framework to compare the directions of movement vectors.

We first confirmed unimodality in the travel headings of our tracked godwits, both by visual inspection and by comparing results from maximum likelihood models of circular orientation developed for animal movement via the *CircMLE* package [45]. Next, we defined two normalized (i.e., direction-only) movement vectors for each location: the total wind flow ($\hat{w}$), and the realized direction of godwit travel to the next location (i.e., the track direction, or where a godwit actually flew; $\hat{r}$). To allow for orientation to a geographic region rather than a particular site, and to account for the fact that the proportion of the flight horizon that encompasses this region will increase with greater proximity, we defined a population-wide *range of preferred directions*. This range was bounded by the western- and easternmost points in the narrowest region of the migratory corridor ($\left[ {\widehat{{d_{w} }},\widehat{{d_{e} }}} \right]$) and had as its center the angular midpoint of this range ($\hat{d}$).

We then calculated the angles separating these normalized vectors (i.e., their “closeness”), focusing on the angles between the realized travel direction and the wind (θ_rw_), the realized travel direction and the midpoint of the preferred range (θ_rd_), the midpoint of the preferred range and the wind (θ_wd_), and the midpoint and edge of the preferred range (θ_dd_), where θ ∈ [θ, π]. To account for imprecision in these measurements, we incorporated two tolerance values: 1) a larger tolerance value (τ_1_ = 0.2 rad or ~ 11.46°) to account for the combined imprecision of the realized and wind directions, and 2) a smaller tolerance value (τ_2_ = 0.1 rad or ~ 5.73°) to account for the imprecision of the realized direction alone, which is generally small for infrequently sampled, fast-flying species ([34]; see Additional file 1 for details on tolerance value estimation). Tolerance values in this way allowed us to account for known and variable imprecision in our data without adding additional unknown sources of imprecision. Together, this information enabled us to differentiate between *fully drifting* flight (realized direction ≈ direction of the wind flow, but not within the preferred range), *supported* flight (realized direction ≈ direction of the wind flow and within the preferred range), and *fully compensating* flight (realized direction ≠ direction of the wind flow and within the preferred range).

**Fig. 2:**
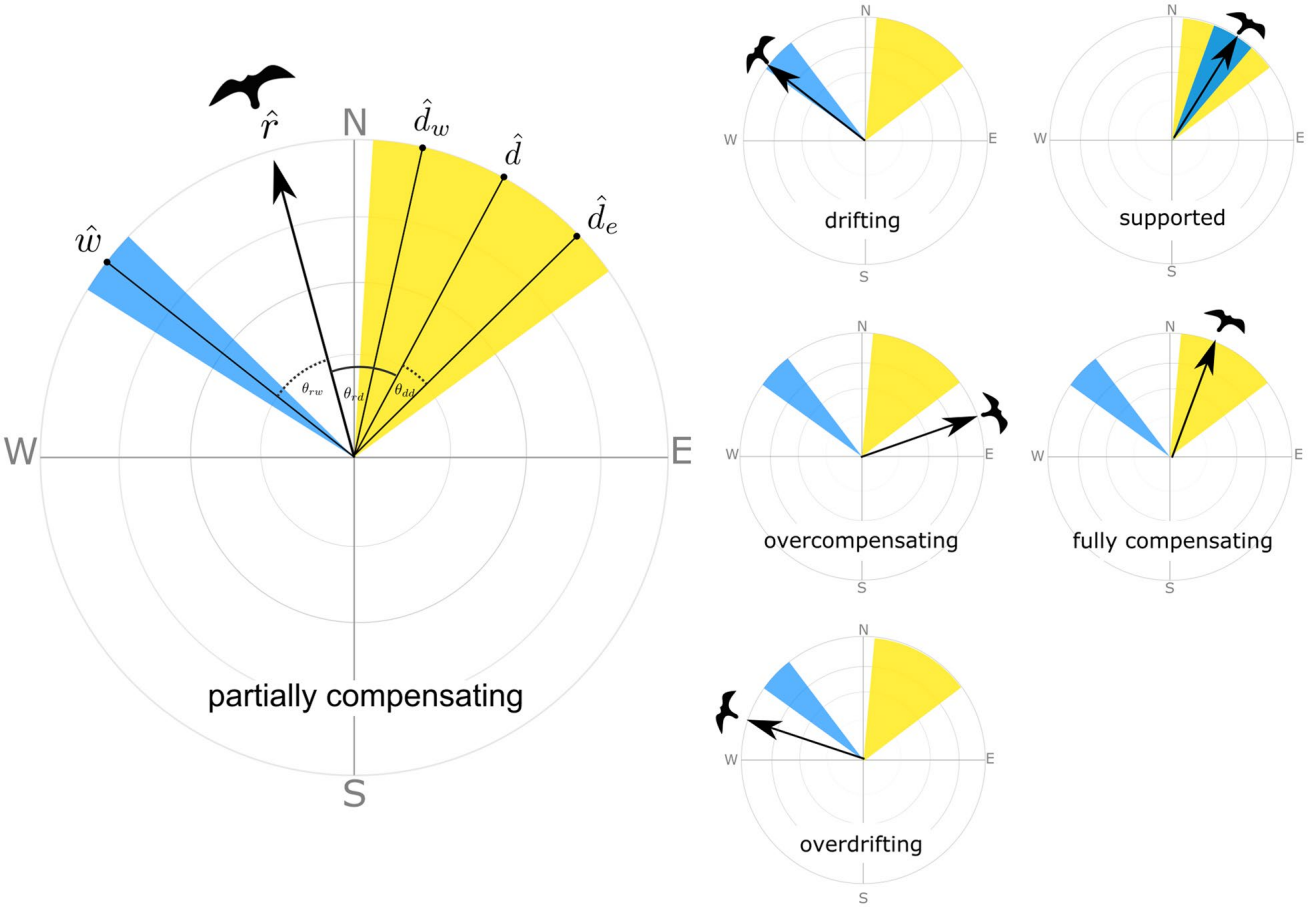
Circular framework for classifying in-flight behaviors using the direction of the wind flow ($\hat{w}$; shown with tolerance value in blue), the range of the preferred direction ($\left[ {\hat{d}_{w} ,\hat{d}_{e} } \right]$; shown with tolerance value in yellow), and the direction of realized movement of the bird ($\hat{r}$; shown as black arrow). Angular differences (θ_rw,_ θ_rd_ θ_dd_) are used to determine the “closeness” of the directions (i.e., if they are approximately similar)

If none of these categories applied, we inspected the arrangement of vectors (i.e., their “betweenness”). The cross products $\left\| {\widehat{r} \times \widehat{d}} \right\|$ and $\left\| {\widehat{w} \times \widehat{d}} \right\|$ returned orthogonal vectors with the sign determined by the degree of rotation from one angle to another. Comparing the signs of cross products thus revealed which vector lay between the other vectors on a semi-circle. Different signs indicated *overcompensating* flight (midpoint of the preferred range lies between the realized direction and wind flow). Similar signs indicated either *partially compensating* flight (realized direction lies between the wind flow and the midpoint of the preferred range) or *overdrifting* flight (defined here as when the wind flow lies between the realized direction and the midpoint of the preferred range). Together, these steps produced an easily implemented, hierarchical decision list that assigned to each in-flight location one prevailing behavior (Fig. 2).

To test our predictions that drift would prevail in the early stages of the flight from Chiloé to the NGC, and particularly over open water, we first examined conditional associations between assigned behaviors and latitudinal bands. We applied an omnibus χ^2^ test for independence and used a *post-hoc* inspection of the adjusted standardized residuals with a Bonferroni adjustment (⍺ = 0.0014, critical value =  ± 2.98; [46] to evaluate cell contributions to significance, using functions from the *stats* [47] and *vcd* [48] packages. Because overdrift was exceptionally rare, we verified that it was distributed across several latitudinal bands and excluded it from the χ^2^ test.

Since categorizing behavior and binning by latitudinal bands may obscure more subtle patterns, we also performed a companion analysis investigating ongoing changes in godwit divergence from the preferred range (θ_rd_) with changes in latitude, wind support, and crosswind using multivariate adaptive regression spline (MARS) models implemented via the *earth* package [49]. MARS models use partitioning and iterative pruning to capture possible interactions and nonlinearities in predictor variables. Because MARS models do not have the ability to deal directly with outliers, we inspected for and removed any extreme outliers (θ_rd_ > 1.5 rad or 90°, *n* = 1) prior to analysis. We centered and standardized our predictor variables prior to inclusion and set *minspan* = 20 to limit over-fitting in sections of the migratory route with few observations. We tested for the possibility of interactive or additive effects and allowed variables to enter linearly or with nonlinear change-points (i.e., hinge functions). Results obtained with MARS and similar machine-learning methods are generally robust to collinearity in the set of predictors [50]; nonetheless, we verified that the variance inflation factors of variables were suitably low (VIF < 2). We report the unscaled, uncentered MARS model parameter estimates.

Finally, to compare our results with patterns observed in another individual tracking study, we adapted methods from Klaassen et al. [13] to evaluate the effect of the forward component of the wind vector in the preferred direction (wind support) on the forward rate of godwit movement in the preferred direction (r_v_), as well as the effect of the lateral component of the wind vector (crosswind) with the lateral rate of godwit movement (r_u_). Individual and year were again included as random effects. We also adapted their calculation for total drift, which is the ratio between the slopes of these models (β_cw_/β_ws_). Residuals from these models informed where godwit lateral movements were most strongly influenced by crosswind.

### Crossing over the Northern Gulf Coast

We predicted that the cumulative effects of wind flow during flight would influence where godwits crossed into North America. To test this, we identified the location of NGC crossover (i.e., crossing over land, but not necessarily stopping) for each complete flight track. When a track contained an onshore location estimate < 10 km from the coast, we used this location. For other tracks, we interpolated a plausible crossover location along the straight-line intersection of the flight track with a coastline shapefile (Natural Earth, 10-m global coastline). For each individual, we calculated its total longitudinal displacement by subtracting its actual longitude at the point of NGC crossover from the crossover longitude of a shortest-distance (i.e., Great Circle) line extending from Caulín on Chiloé to the geographic midpoint of the preferred range. We used a linear regression to assess the extent to which this displacement was shaped by mean crosswinds aggregated over all preceding locations along the flight track. To control for displacement associated with habitat selection during unexpected stopover events in Central America, individuals with complete tracks that stopped prior to the NGC (*n* = 3) were removed from this analysis.

We present results as means ± standard deviations or medians and interquartile ranges, unless otherwise stated. The global significance of the fixed effects in all models was approximated with parametric bootstrapped *p* values (1000 simulations) obtained with the package *afex* [51]. We evaluated the uncertainty of parameter estimates using 95% parametric bootstrapped confidence intervals (1000 simulations) obtained with the ‘confint.merMod’ function in *lme4* [40]. Estimations of the partial variance explained by fixed effects (*R*^*2*^_*LMM(f)*_) were calculated using ‘rsq.lmm’ function via the *rsq* package [52]. Random intercepts for year and individual were retained if their variance contributions were greater than (near) 0 and fits did not result in singularity or non-convergence [53]. All analyses were conducted in the R programming environment, version 4.0.3 [47].

## Results

From 2019–2021 we successfully recorded 24 complete and 5 partial northward migratory tracks from Chiloé to the NGC. These included repeat tracks from two individuals, which were followed for three years. Our sample size was reduced by individuals that deferred migration and oversummered in Argentina (*n* = 8), a behavior known to regularly occur in godwits [54]. Device loss and device malfunction, as confirmed by subsequent resightings of individuals on Chiloé, also reduced our sample, and mortality events likely also played a role. Of the godwits included in this study, morphological characteristics and molecular sexing [26] determined that the group consisted of 14 males and 11 females. We included partial tracks in our analyses as we saw no indications of erratic or unexpected in-flight behavior prior to transmitter failure.

Nearly all tracked godwits departed on a schedule associated with Alaskan breeding populations, leaving Chiloé in early- to mid-April over a span of 7 ± 1 d. One godwit migrated to Hudson Bay and regularly departed 2–3 weeks later. In 2019, this individual departed Chiloé and flew well within the migratory corridor of Alaska-breeding conspecifics, diverging only after NGC crossover; we therefore included this track for in-flight behavioral analyses, but excluded its post-NGC movements from estimation of corridor width. Since this individual did not return to Chiloé before migrating northward in 2020, its track from that year was excluded from analyses.

Godwits with complete tracks undertook continuous flights lasting on average 5.98 ± 0.81 d and covering 8,361 ± 671 km before making their first stops. In nearly all tracks, godwits did not stop until after arriving on the NGC; however, 3 tracks contained unexpected early stops along the flight path in Central America, which were removed from behavioral analysis.

### Experienced wind conditions

Godwit ground speeds were best predicted by a strategy in which individuals flew at the altitude offering optimal (maximum) wind support in the preferred direction of movement, but were restricted to altitudes at or below 3000 m.a.s.l. This model was a reasonable fit for the data (*R*^*2*^_*LMM(f)*_ = 0.371, *p* < 0.001), predicting plausible godwit airspeeds of 12.70 ms^−1^ (95%CI: 12.14, 13.27). Among-individual variation in the intercept was minimal (σ_ID_ = 0.380; σ_LMM_ = 3.800). The next best model (∆AIC_c_ = 9.42, Akaike weight = 0.009) described a similar strategy but also allowed selection of two higher altitudes (see Additional file 1: Table 1). In both models, however, godwits most often flew at the lowest altitudes (100 or 750 m.a.s.l.), particularly during flights over the Pacific Ocean (74.5% of locations in the top model). While we found that wind conditions differed among altitudes, there was often little difference between conditions at the altitude offering the maximum wind support and conditions at the next-best altitude. Wind directions at these altitudes were strongly correlated (r = 0.62, *p* < 0.001), with a median difference of 8.95° (IQR 23.42°), as were wind speeds (r = 0.86, 95%CI: 0.83, 0.87, *p* < 0.001).

**Fig. 3:**
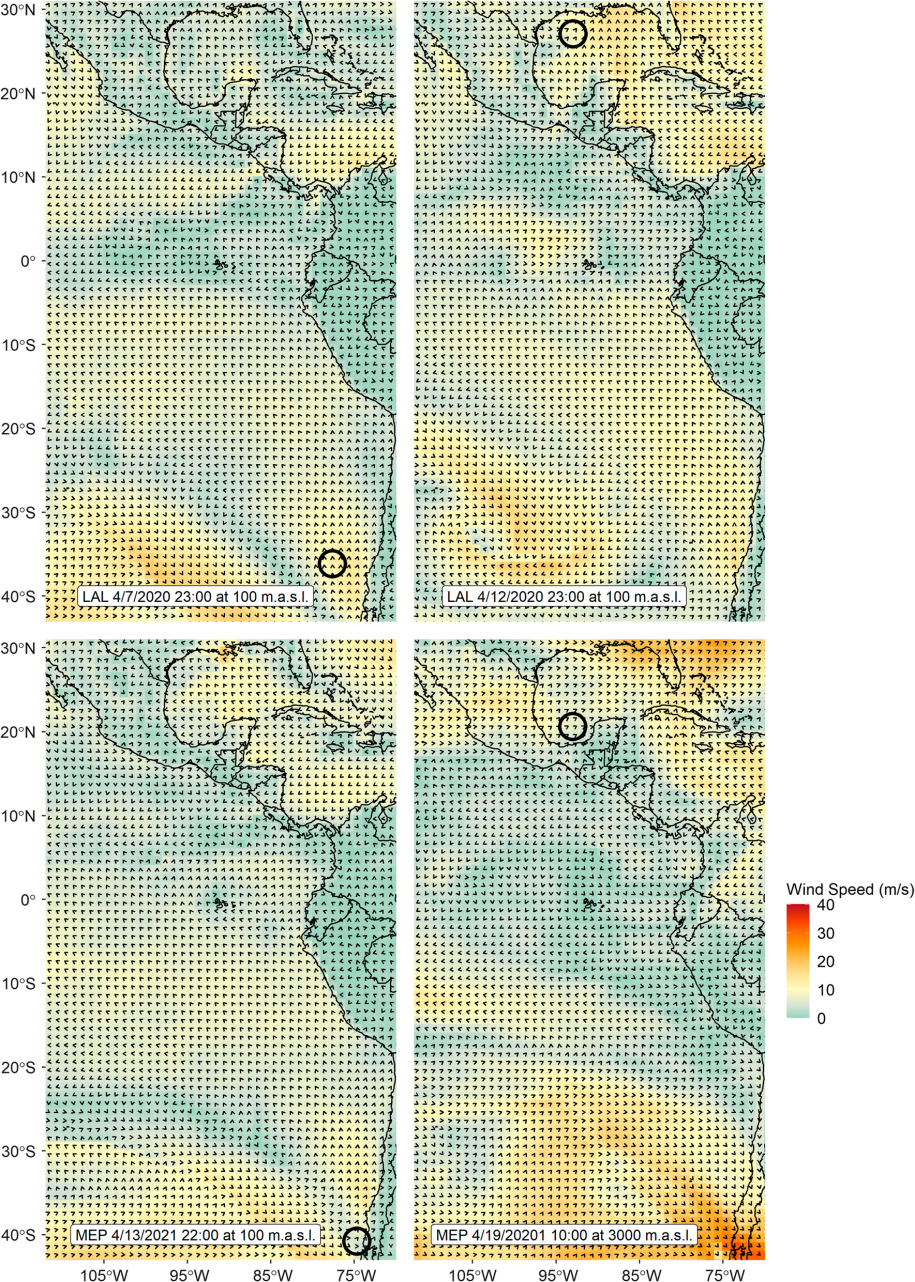
Wind conditions during flight for two godwits (‘LAL’ and ‘MEP’). Arrows reflect direction of the wind flow at the time and predicted altitude (in meters above sea level) corresponding with a godwit location (shown as black circle). Wind conditions and godwit locations are resampled to a 120-km grid for ease of visualization. Dates are in month/day/year format and times are in UTC

After departure from Chiloé, godwits experienced variable wind conditions (Fig. 3), including generally high scalar mean wind speeds ($\overline{x}$ = 9.58 ± 3.45 ms^−1^) concentrated around a northward directional peak ($\overline{\theta }$ = 357.56°, $\overline{R}$ = 0.928) from 43 to 30°S (Table 1). Wind speeds remained high and directionally concentrated until the equator, followed by decreases in speed and increases in directional dispersion from 0 to 20°N. From 20 to 30°N, a band which encompassed the approach to the NGC, godwits encountered directionally dispersed wind flow and high mean wind speeds ($\overline{x}$ = 9.16 ± 4.90 ms^−1^, $\overline{\theta }$ = 348.41°, $\overline{R}$ = 0.597). Rayleigh tests confirmed the non-uniformity of wind direction in all bands (*p* < 0.001).

**Table 1:** Wind conditions and godwit movement along the migratory corridor

Latitudes	Wind $\overline{\theta }$ ± sd (°)	Wind $\overline{R}$	Wind speed $\overline{x}$ ± sd (ms^−1^)	Godwit $\overline{\theta }$ ± sd (°)	Godwit $\overline{R}$	Movement residual
43–30°S	357.56 ± 0.39	0.928	9.58 ± 3.45	340.05 ± 0.12	0.993	− 1.46
30–20°S	331.44 ± 0.39	0.928	8.98 ± 2.47	337.19 ± 0.17	0.985	− 1.82
20–10°S	314.59 ± 0.25	0.970	8.56 ± 2.58	343.22 ± 0.16	0.987	0.39
10°S–0°	321.15 ± 0.40	0.925	6.01 ± 2.24	344.39 ± 0.27	0.965	0.09
0–10°N	348.31 ± 0.99	0.611	3.99 ± 2.08	353.02 ± 0.30	0.955	1.80
10–20°N	311.41 ± 1.14	0.523	4.64 ± 3.61	341.22 ± 0.34	0.945	− 0.03
20–30°N	348.41 ± 1.01	0.597	9.16 ± 4.90	337.19 ± 0.31	0.954	− 2.13

Circular mean wind flow directions ($\overline{\theta }$), resultant lengths ($\overline{R}$), and scalar wind speeds ($\overline{x}$) along the first stage of northward migratory flight, as well as associated statistics for godwit realized travel direction. Godwit preferred direction is approximately 340.97° along this route. Residuals reflect LMM for effect of crosswind on godwit lateral movement (see Additional file 1: Table S3).

### In-flight behavior

Tracked godwits traveled along a mean realized direction of 343.79 ± 0.29°, a close match to the bearing of a shortest-distance (i.e., Great Circle) line from departure to the geographic midpoint of the narrowest region of the migratory corridor (340.98°). Collectively their orientations during this stage were unimodal, as confirmed by maximum likelihood model selection (ΔAIC_c_ = 395.15).

**Fig. 4:**
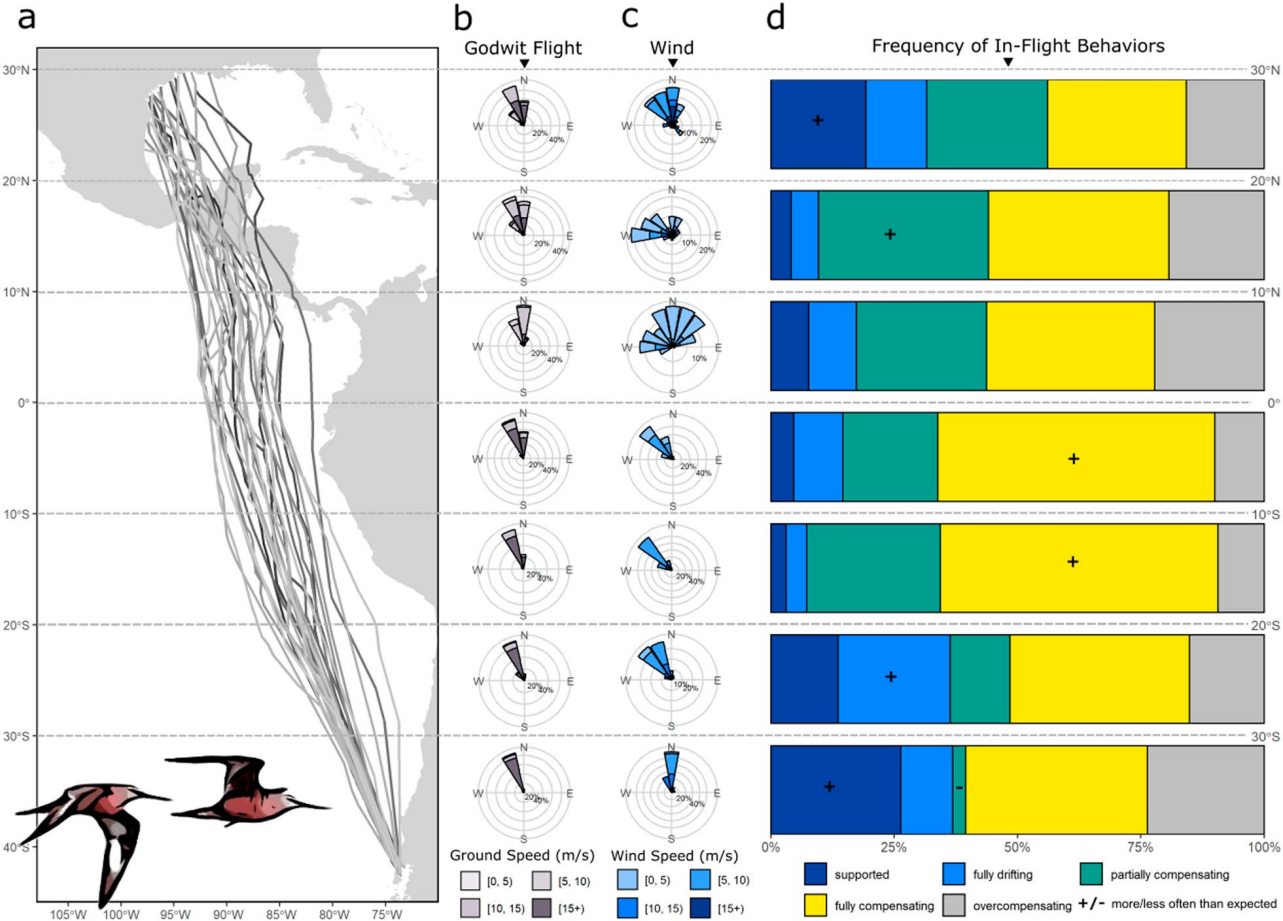
Godwit flight in relation to wind flow during an initial bout of migratory flight, aggregated by ~ 10° latitude. **a** Flight tracks depicted as lines extending from departure sites on Chiloé Island, Chile to arrival on the northern coast of the Gulf of Mexico. **b** Godwit realized travel direction and speed. **c** Wind direction and speed. **d** Frequency of in-flight behaviors (supported, full drift, partial compensation, full compensation, and correcting). Behaviors denoted with (+) occurred more often than expected and (−) occurred less often than expected via χ^2^ test. For circular plots, directions are depicted in bins of 22.5° for visualization, with bar length proportional to the frequency of observations and bar color representative of speed

Our circular framework identified six in-flight behaviors, which varied in frequency along the migratory corridor (Fig. 4). Full compensation was the most frequent behavior, accounting for 41.1% of all observed flight segments (*n* = 283). Fewer segments were associated with partial compensation (23.5%), supported flight (8.1%), full drifting (9.4%), or overcompensation (16.0%), while overdrift was rare (1.9%). The prevalence of full compensation remained constant across wind conditions. For example, full compensation was the dominant behavior under crosswinds to the east (32.0%) and west (45.3%) of the midpoint of the preferred range and under various levels of wind support, including low (ws < 2.05 ms^−1^, equal to 25th percentile; 37.2%), mid (2.05 ms^−1^ < ws < 6.69 ms^−1^, equal to IQR; 45.3%), and high levels (ws > 6.69 ms^−1^, equal to 75th percentile; 36.4%). Full compensation was also prevalent across regions, comprising the largest proportion of behaviors exhibited over the Pacific Ocean (45.1%), Central America (23.9%), and the Gulf of Mexico (31.4%). Finally, we also verified that movements classified as full compensation were not merely cases in which the wind flow was nearly aligned with the preferred range (median θ_rw_ = 0.56 radians or 31.89°; Additional file 1: Fig. S3).

Our χ^2^ test revealed several weak associations between behavior and latitudinal bands (χ^2^(24, *n* = 676) = 100.06, *p* < 0.001, *ɸ*_*c*_ = 0.192). After departure (43–30°S), godwits followed supportive winds more often than expected and partially compensated less than expected. Full drift from 30 to 20°S and full compensation from 20°S to 0° also occurred more than expected, as did partial compensation while crossing Central America (10–20°N). No behaviors exhibited a significant departure from expected frequencies in the band approaching Central America (0–10°N), while supported flight occurred more often than expected over the Gulf of Mexico (20–30°N). Sensitivity tests for behavioral classification showed that allowing godwits to select the altitude with the second highest (rather than highest) wind support altered the behavior in some cases (*n* = 130 of 674 locations), but our general results remained robust; only the significant departures of fully compensating flight in 20–10°S and supported flight in 20–30°N were lost.

**Fig. 5:**
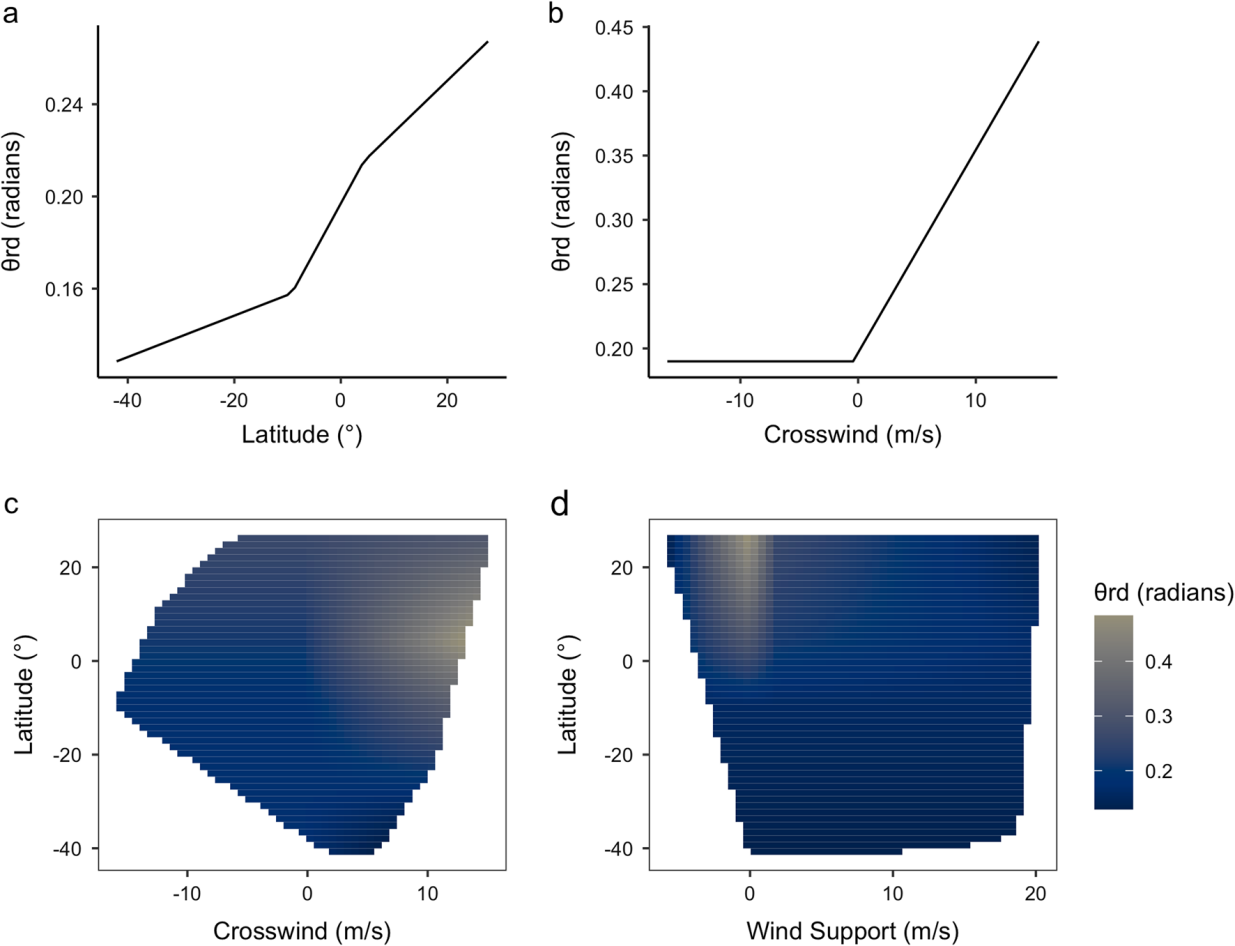
Partial dependence plots depicting change in θ_rd_ (angular difference between godwit realized travel direction and midpoint of the preferred range) along the first flight bout with **a** change in latitude, **b** changes in crosswind, **c** interactive effect of latitude and crosswind; and **d** interactive effect of latitude and wind support. For interaction plots, values are restricted to within a convex hull of training values (i.e., no extrapolation beyond study data). Variables and hinge functions selected by the MARS process

To model behavioral change in a continuous framework, the MARS procedure selected (in order of importance) latitude, wind support, and crosswind, with interaction terms retained for latitude × crosswinds and latitude × wind support. Change-points were located at latitudes of 9.21°S and 4.32°N, wind supports of − 0.031 ms^−1^ and 1.91 ms^−1^, and a crosswind of − 0.43 ms^−1^ (see Additional file 1: Table 2, Fig. 5). The best MARS model (*GRsq* = 0.110, *R*^2^_MARS_ = 0.155) was an improvement on a standard linear model (*R*^2^_LM_ = 0.071), though most variation in θ_rd_ still remained unexplained.

Using methods from Klaassen et al. [13], we confirmed that godwit forward and lateral movements were affected by winds along the route (see Additional file 1: Table 3). For each 1 ms^−1^ increase in wind support (i.e., tailwind), godwits traveled 0.799 ms^−1^ faster (95%CI: 0.721, 0.877; *R*^2^_LMM(f)_ = 0.383, *p* < 0.001), with significant random effects of individual and year (σ_ID_ = 0.374, σ_year_ = 0.730, σ_LMM_ = 3.800). The effect was likewise positive but weaker for crosswinds (*R*^2^_LMM(f)_ = 0.096, *p* < 0.001), where for each 1 ms^−1^ increase in crosswind in either direction, godwits traveled 0.315 ms^−1^ faster in the same direction (95%CI: 0.241, 0.393). This varied for individuals (σ_ID_ = 0.645, σ_LMM_ = 3.371), but not across years. With this model, the largest mean residual of lateral movement occurred from 20 to 30°N (Table 1). Overall, godwits allowed approximately 39.4% drift over the course of their flight. However, the locations of NGC crossover demonstrated no significant association with overall mean crosswinds (*R*^*2*^ = 0.045, *p* = 0.192).

## Discussion

Our study tracked the in-flight behavior of individual Hudsonian godwits in relation to wind flow over the course of an extreme long-distance, transoceanic flight. Godwits encountered changing but generally favorable wind conditions along the way. While drifting was not uncommon at the beginning of this flight, we nonetheless found consistently high rates of full compensation, even over open ocean, complicating the classical ‘drift first, compensate later’ prediction for migratory flights [6] and the presumed reliance on visual landmarks [20]. We also failed to find evidence that cumulative crosswinds determined where godwits crossed the coastline along the Northern Gulf of Mexico at the end of this flight. These results reinforce behavioral complexity in response to wind flow in other migratory species [13], but in a markedly different flight type: one traversing vast expanses of open ocean, and one in which lift must be sustained for more than five days by energetically-demanding flapping flight. Our results thus suggest that for godwits and other migrants that cross similar barriers, frequent compensation may be necessary to minimize the risk of costly and perilous displacements.

### Accurately classifying in-flight behaviors

A lack of precise data for migratory flights has long constrained our ability to infer avian behavior in response to dynamic aerial environments. Most tracking devices that are sufficiently lightweight for shorebirds and passerines do not have the ability to measure an individual’s exact flight altitude, and those that do often provide measurements with considerable error—particularly for transmitters with large reporting intervals [55]. Inferences about wind use and preferred flight heights are therefore limited [30]. Similarly, because most devices do not measure instantaneous heading, estimates of preferred direction can be difficult. Our circular framework suggests that these limitations can be overcome and that meaningful analyses of in-flight behavior using individual tracking data are possible, even for migratory flights that involve directional shifts and flexible intermediary destinations.

We estimated conditions aloft for godwits using a combination of altitude-dependent wind conditions, godwit ground speeds, and known behavioral trends in similar migratory species. We found that godwits generally appeared to seek out favorable wind conditions in their preferred direction within a limited vertical column extending from 100 to 3000 m.a.s.l. This often entailed low-level flights over the Pacific Ocean, a strategy that has been documented in other shorebirds making transoceanic crossings [56], as well as dynamic altitude-switching, which may incur lower costs for nimble, long-winged godwits than for other larger-bodied species [57]. Other factors not included in our models, such as predation risk or thermoregulation, may have also influenced flight altitude [58, 59], though our models suggest that their effects are likely minimal over much of this flight. Strong correlations between wind conditions at two favorable altitudes also underscore that we need not know the precise altitude at which a godwit was flying to approximate the wind conditions it was experiencing and classify its behavior.

Using wind conditions derived from these altitudes, our circular framework differentiated six in-flight behaviors. While these behaviors may be conflated or misclassified by frameworks that assume an individual is navigating directly toward a single site, we used a broader *range of preferred directions* that accommodates interindividual variability/flexibility and the ecological bounds of the migratory corridor. This range is also more representative of the in-flight experience, as it broadens with increasing proximity to the intended destination just as it does for birds on the move (see Additional file 1: Fig. S2). Our framework is also highly modifiable, allowing us to account for known and variable imprecision in our data, and it may be useful for other in-flight behavioral studies with flexible destinations.

### Drift first, compensate later?

We found little support for our prediction that godwits would tolerate drift early in their flight and gradually begin to increase compensation as they approached North America. Instead, both fully supported flight and full compensation were common immediately after leaving Chiloé. This suggests that godwits—like other transoceanic migratory birds [30]—may sometimes select favorable winds for departure, but that they are capable of departing under suboptimal conditions [12]. We also found that fully compensating was the most frequent behavioral response along the entirety of their flight and was no less frequent in the earliest stage of their flight than it was several days later, as godwits approached North America. In fact, as distance from the departure site increased, individuals slightly increased their angular discrepancy from the midpoint of the preferred range, with an even more pronounced increase (i.e., a relaxation in compensation) north of 9.21°S. Notably, we did not see evidence that this pattern was the result of wind flow nearly aligning with the preferred direction of travel. While godwits generally migrated in prevailing tailwinds, these winds were often directed more than 20° away from their preferred direction of travel and full compensation dominated godwit behavior under a variety of wind directions and wind speeds. Overall, we found that godwits allowed less total drift (39%) during this initial flight than observed in the entire trajectory of raptors crossing terrestrial landscapes (47% average; [13]), despite the fact that godwits flew continuously for more than five days without resting or refueling.

It is important to note that the full godwit migratory route may indeed entail increasing rates of full compensation. Nonetheless, while we did not examine later flights over land, fully compensating did not merely become important later in the journey for godwits. This pattern may, in part, reflect the wind conditions that our tracked godwits experienced as they flew. However, we also found that godwits did not appear to be responding to conditions in the same way throughout the flight. Rather, their responses to winds changed with their positions in geographic space. For example, godwits generally flew north and traveled within or near their preferred range, but we found that θ_rd_ was often more strongly limited for crosswinds flowing to the west (cw < 0). The west side of the migratory corridor thus appeared to constitute a hard “edge,” particularly over the Pacific Ocean: godwits strongly corrected for displacement to the west, even when crosswinds were strong. This strategy was likely shaped by the consequences of displacement over the vast Central Pacific Basin, which could entail morality by energetic depletion, eventual stranding in Oceania where global wind patterns preclude return, or a more westerly route through the arid central-western region of North America, where wetland stopover sites are scarce. At low latitudes, godwits additionally limited their displacement by crosswinds to the east (cw > 0), suggesting that the arid coast of South America—which may likewise constrain fueling—may also be a hard edge. Both edges ‘softened’ in the approach to Central America, as individuals allowed more divergence from the preferred direction with increasing proximity to land. Wind support also displayed an interactive effect with latitude: when available wind support was low and godwits were nearing Central America, they diverged more from the preferred direction, again suggesting a relaxation of risk with proximity to land. Thus, behavior during transoceanic flights may be better understood as a reflection of both wind conditions and perceived risk. That godwits perceive risk differently in different places further underscores that many long-distance migratory birds are likely employing a sophisticated positional orientation (i.e., “map-sense”) as they travel [60].

### Behavior over open ocean

We found no evidence to support our prediction that compensation is nonadaptively limited in the absence of topographical landmarks. In fact, full compensation was especially pronounced from 20°S to 0°, which includes the most remote regions of the Pacific Ocean along the godwit migratory corridor. To our knowledge, the only salient visual landmarks here are at the northern edge of this region (e.g., Galapagos Islands), yet the fact that many godwits did not pass near or over the islands suggests that godwits are capable of compensating without the aid of topographic features.

As remarkable as this capacity may seem, it may be critical for the survival of long-distance aerial migrants. For godwits, compensation from 20°S to 0° broadly corresponds with the northward extent of the counter-clockwise rotation of the SPSA [61], which carries godwits north—often in the preferred range—after departure from Chiloé, but which poses a serious threat if followed too far and too long. This raises the question of how godwits estimate both their geographic position and their rate of movement in order to pull out of this rotation at the appropriate time. The South Pacific Ocean is not without visual cues; some of our tracked godwits, for example, may have used the appearance of the Galapagos Islands as a signpost of the western corridor boundary or a signal of the approach of Central America. The broad prevalence of compensation, however, suggests that other sensory information, such as shifts in temperature or humidity that mark passage through broad wind regimes, celestial [62] or magnetic cues [24], or surface swell patterns [6] could assist in position and rate estimation over open ocean, and integrating multiple cues may be more effective than relying on a single cue alone [63]. Whatever the mechanisms, godwits join other birds—including homing seabirds [17, 64] and migrating juvenile osprey (*Pandion haliaetus*) [24]—in revealing the ability to compensate over open ocean.

### Cumulative wind effects on crossover

Finally, we did not find support for our prediction that cumulative crosswinds experienced over the course of a long-distance, non-stop flight would affect an individual’s location days later, when it crossed into North America. This is consistent with our finding that full compensation was generally common but increased in frequency in some geographic regions (20°S–0°); crosswinds, therefore, may have displaced godwits less in these regions than in others. Considerable behavioral variation over the Gulf of Mexico also suggests that godwits are not only encountering a wide range of wind conditions, but that other factors in the final hours of flight—such as remaining fuel levels after several days of continuous flight—may also have influenced where godwits crossed the NGC. Individuals with severely depleted stores, for example, may have been more driven to reach the nearest land than to minimize time or distance. Indeed, we observed several abrupt westward turns (*n* = 3) over the Gulf of Mexico that could not be explained by improvements in wind support or realignment with the preferred range and may instead represent emergency maneuvers to locate the nearest land. Behavioral variation may also be driven by tendencies in some individuals to navigate to familiar places along the NGC, though we did not see consistent evidence for this in our data. While one individual tracked repeatedly over three years (‘KCV’; see Additional file 1: Fig. S1) generally crossed the NGC in central Texas, it did not always stop there. Another individual (‘KCL’) also tracked for three years did not consistently return to one region of the NGC, converging on similar interannual routes only after crossing over land.

Intriguingly, individual differences were evident in all our mixed models, though their contributions to variation were small. These differences could be a result of differing levels of experience. Just as juveniles and adults behave differently during migration [65, 66], older adult godwits may have accrued more experience over multiple migratory flights that enables them (or groups which they lead) to travel more efficiently [67], and perhaps compensate more consistently and correctly [17], than younger godwits. Competitive ability may also confer higher pre-departure fuel loads [68] that better facilitate compensating behavior for some individuals, particularly later in the flight. Studies like ours that affix transmitters in advance of migration often lack direct information on departure fuel loads, but individual consistency in oxidative balance may offer a future avenue for investigating these differences [69].

## Conclusions

We found that fully compensating for wind displacement was a critical strategy for godwits making a long-distance, transoceanic flight. While godwits often followed wind flow in the early stages of this journey, they nonetheless engaged in full compensation more frequently than any other behavior during the entirety of the flight, including during passage over an oceanic landscape that seemingly lacked topographic landmarks. We found that the rate of compensation varied with proximity to land, suggesting that godwits, like raptors, respond differently to wind resistance depending on their location in geographic space [13]. Our results emphasize the tremendous physiological and navigational capabilities required to safely complete barrier-crossing migratory flights, which may offer considerable advantages in time and energy savings or predator avoidance [56], but which nonetheless require dynamic responses to changing levels of resistance (due to wind conditions) and risk (due to geographic context) along the way.

Looking ahead, our work raises questions about how changing wind regimes may impact the flights of long-distance aerial migrants [70]. The shifting center of the SPCA is expected to bring increased wind speeds off the coast of Chiloé [71], potentially supporting less costly departures if wind direction remains stable, but corresponding downstream changes from 20°S to 0° could dramatically increase the risk of westward displacement and/or the energy required to reach their first stopover sites. With few alternative routes, how well godwits cope with these changes will therefore have profound influences on their future population dynamics. Predicting large-scale changes in wind regimes, as well as the extent to which aerial migrants may adjust their behavior in response to increases in resistance and risk, may be more critical for long-term conservation efforts than currently recognized [72].

## Supplementary Information

**Additional file 1: Methods**—Procedural information for defining the migratory corridor, details on tolerance value selection, and R code for behavioral analyses. **Results**—**Table S1**: Results of LMMs evaluating the effect of wind support on measured ground speeds. **Table S2**: Model estimates for angular difference between realized and preferred directions in godwit northward migration. **Table S3**: Results of LMMs for godwit forward and lateral movement as a result of wind support and crosswind, respectively. **Fig. S1**: Movement tracks of individuals followed repeatedly over three years. **Fig. S2**: Comparison of behavioral classifications using a single preferred direction versus a range of the preferred direction. **Fig. S3**: For movements classified as fully compensating, godwit divergence from the preferred direction at varying wind speeds.

## Data Availability

The tracking data used in this study are stored in Movebank (movebank.org, study name “Migrations of Hudsonian godwits”) and will be available in the Movebank Data Repository (https://doi.org/10.5441/001/1.t81488n5) after a one-year embargo, or on request. Wind data were downloaded from the Copernicus Climate Change Service (C3S) Climate Data Store (CDS) (https://doi.org/10.24381/cds.bd0915c6).

## Abbreviations

NGOM: Northern coast of the Gulf of Mexico
SPSA: South Pacific subtropical anticyclone
ITCZ: Intertropical convergence zone
ws: Wind support
cw: Crosswind
