# Supplementary material for: Compensation for wind drift prevails for a shorebird on a long-distance, transoceanic flight
Source: Mov Ecol. 2022 Mar 7;10:11. doi: 10.1186/s40462-022-00310-z (PMC8900403; doi:10.1186/s40462-022-00310-z)
Supplement: Supplementary file 1 — Additional file 1: Methods—Procedural information for defining the migratory corridor, details on tolerance value selection, and R code for behavioral analyses. Results—Table S1: Results of LMMs evaluating the effect of wind support on measured ground speeds. Table S2: Model estimates for angular difference between realized and preferred directions in godwit northward migration. Table S3: Results of LMMs for godwit forward and lateral movement as a result of wind support and crosswind, respectively. Fig. S1: Movement tracks of individuals followed repeatedly over three years. Fig. S2: Comparison of behavioral classifications using a single preferred direction versus a range of the preferred direction. Fig. S3: For movements classified as fully compensating, godwit divergence from the preferred direction at varying wind speeds. [file 40462_2022_310_MOESM1_ESM.pdf]

Additional File 1

***for* Compensation for wind drift prevails for a shorebird on a long-distance, transoceanic flight**

Jennifer A. Linscott, Juan G. Navedo, Sarah J. Clements, Jason P. Loghry, Jorge Ruiz, Bart M. Ballard, Mitch D. Weegman, and Nathan R. Senner

Methods

Defining the Aerial Migratory Corridor

To establish the preferred migratory direction for our tracked godwits, we adapted methodology from Pearse et al. (2018) [1] to define their migratory corridor. We began with all godwit locations associated with northward migration. Overland locations where ground speeds fell below 3 ms^{-1} [2] and consecutive movements traversed < 15 km were grouped into discrete stopover events; we retained only the first and last locations for each stopover and retained all other locations. When directional movement ceased or stabilized for longer than 14 days—a pattern indicating arrival at the breeding grounds, mortality, or transmitter failure—we considered this to be the end of northward migration and removed all associated locations.

Next, we created a 95% core area polygon to serve as the migratory corridor. As we were primarily interested in the first stage of migration (i.e., from departure through the main stopover region), we focused on the area south of 50°N. We divided this area into 'windows' of equal height (see methodology below) and calculated window vertices as the medians of the transmitter-reported locations' y-dimensions (i.e., north/south) and percentiles of the locations' x-dimension (i.e., east/west) corresponding to the area between the 2.5th and 97.5th percentiles (n = 1,811 locations). We then connected the vertices to form a polygon outlining the migratory corridor.

To determine the optimal spacing for polygon vertices—in essence, one that sufficiently captured both within and among individual variation while avoiding overfitting—we tested multiple window heights from 2° to 6° latitude in 1° increments. We used 1,000 bootstrap estimates to compare how well the resulting polygons fit our transmitter-reported locations, seeking to maximize this value. We then used the fractal dimension index via the *landscapemetrics* package in R [3] to compare the complexity of the resulting polygons; as lower values were indicative of less-complex shapes, we sought to minimize this value. These comparisons informed our selection of the best-fitting, optimally smoothed polygon to serve as the overall migratory corridor for our tracked godwits.

Tolerance Value Calculation

Behaviors such as full compensation, full drift, and supported flight occur when two or more critical movement vectors are aligned. However, alignment need not be perfect, particularly given the error inherent in vector measurements. To capture cases in which these vectors are nearly aligned, we used tolerance values derived from the known imprecision of our vector estimates. When evaluating if a godwit's realized travel direction is aligned with its preferred direction, we allowed vectors to be separated by up to 0.1 rad or ~5.73°. We estimated an appropriate value using the arctangent of the maximum location error (13.0 km, corresponding with the 95th error percentile of the least-precise location classes included in our analysis; [4]) divided by the median distance traveled between locations in our filtered, thinned flight tracks (129 km). When evaluating if a godwit's realized direction is aligned with the wind direction, we allowed vectors to be separated by up to 0.2 rad or ~11.46°, a larger value reflecting imprecision both in the godwit locations and in altitude estimation. We estimated an appropriate value using the sum of the smaller tolerance value (0.1 rad) and median difference between wind directions at the optimal and second-optimal altitude (~0.16 rad). Tolerance values can be easily adjusted for more or less precise data sources.

R Code for Behavioral Assignments

We classified behaviors by evaluating the angles between three normalized movement vectors: the angle between subsequent realized travel direction and wind direction (θ_{rw}), the angle between realized travel direction and the midpoint of the preferred range (θ_{rd}), and the midpoint of the preferred range and the wind direction (θ_{wd}), and the midpoint and one edge of the preferred range (θ_{dd}) at each location. The following hierarchical decision list assigned one behavior to each location:

If $\theta_{rw} < \tau_1$ & $\theta_{rd} > (\theta_{dd} + \tau_2)$, then *fully drifting*.

If $\theta_{rw} < \tau_1$ & $\theta_{rd} < (\theta_{dd} + \tau_2)$, then *supported*.

If $\theta_{rw} > \tau_1$ & $\theta_{rd} < (\theta_{dd} + \tau_2)$, then *fully compensating*.

If $|| \hat{r} x\ \hat{d} || * || \hat{w} x\ \hat{d} || < 0$, then *overcompensating*.

If $|| \hat{r} x\ \hat{d} || * || \hat{w} x\ \widehat{d} || > 0$ & $\theta_{rd} < \theta_{wd}$, then *partially compensating*.

If $|| \hat{r} x\ \widehat{d} || * || \hat{w} x\ \hat{d} || > 0$ & $\theta_{rd} > \theta_{wd}$, then *overdrifting*.

Code for implementation of this list in R is given below:

```
library(geosphere)
library(dplyr)
library(circular)

# generate a sample data frame
# observations include the date/time of each location, location coordinates,
# heading to the next location (in radians), and wind direction (in radians)

df <- data.frame(datetime = c("2019-04-13 09:00:00", "2019-04-13 18:00:00"),
                 lon = c(-84.93930, -84.73811),
                 lat = c(2.940531, 5.2316542),
                 travel_dir_rad = c(0.08782884, 5.92948204),
                 wind_dir_rad = c(0.1114553, 0.2243227))


# establish the boundaries of the preferred direction

westernmost <- c(-99.35043, 37.031573)
easternmost <- c(-95.39522, 37.031573)

# for each location, calculate the heading to the boundaries of
# the preferred range

df <- df %>%
       rowwise() %>%
       mutate(angle2westernmost = geosphere::bearing(c(lon, lat), westernmost),
             angle2easternmost = geosphere::bearing(c(lon, lat), easternmost))

# convert the heading (-180, 180) to radians

df <- df %>%
       mutate(angle2west_deg = (angle2westernmost + 360) %% 360,
             angle2east_deg = (angle2easternmost + 360) %% 360)
```

```
df <- df %>%
       mutate(angle2west_rad = rad(angle2west_deg),
              angle2east_rad = rad(angle2east_deg))


# calculate the midpoint of the preferred range

angle_avg <- function(angle1, angle2) {
  a <- ifelse(angle1 > angle2, angle2, angle1)
  b <- ifelse(angle1 > angle2, angle1, angle2)
  c <- ifelse(((b - a) > rad(180)), b-rad(360), b)
  d <- (c + a)/2
  return(d)
}

df <- df %>%
       mutate(goal_mid_rad = angle_avg(angle2east_rad, angle2west_rad))


# calculate normalized movement vectors

df <- df %>%
  mutate(track_x = cos((2*pi)-travel_dir_rad),
         track_y = sin((2*pi)-travel_dir_rad),
         wind_x = cos((2*pi)-wind_dir_rad),
         wind_y = sin((2*pi)-wind_dir_rad),
         goal_east_x = cos((2*pi)-angle2east_rad),
         goal_east_y = sin((2*pi)-angle2east_rad),
         goal_mid_x = cos((2*pi)-goal_mid_rad),
         goal_mid_y = sin((2*pi)-goal_mid_rad))

# determine angles between normalized vectors

df <- df %>%
  mutate(track_wind = acos((track_x*wind_x) + (track_y*wind_y)),
         track_goal = acos((track_x*goal_mid_x) + (track_y*goal_mid_y)),
         goal_edge = acos((goal_mid_x*goal_east_x) + (goal_mid_y*goal_east_y)),
         goal_wind = acos((goal_mid_x*wind_x) + (goal_mid_y*wind_y)),
         cross_track_goal = (track_x*goal_mid_y)-(track_y*goal_mid_x),
         cross_wind_goal = (wind_x*goal_mid_y) - (wind_y*goal_mid_x),
         cross_prod = cross_track_goal*cross_wind_goal)

# set rules for behavioral assignment

df <- df %>% mutate(behavior = case_when(
  track_wind < 0.2 & track_goal > (goal_edge + 0.1) ~ "drift",
  track_wind < 0.2 & track_goal < (goal_edge + 0.1) ~ "supportive",
  track_wind > 0.2 & track_goal < (goal_edge + 0.1) ~ "compensation",
  cross_prod < 0 ~ "overcompensation",
  cross_prod > 0 & track_goal < goal_wind ~ "partial",
  cross_prod > 0 & track_goal > goal_wind ~ "overdrift",
))

# visualize the results

vecs <- match(c("travel_dir_rad", "wind_dir_rad",
             "angle2east_rad", "angle2west_rad"), names(df))
df2 <- df[,c(vecs)]
df2 <- gather(df2, key = "type", value = "angle")
df_circ <- circular::circular(df2$angle, units = "radians", template =
"geographics")

track <- df_circ[1]
```

```
wind <- df_circ[3]
east_goal <- df_circ[5]
west_goal <- df_circ[7]


plot.circular(df_circ)
arrows.circular(track, col = "red")
arrows.circular(wind, col = "blue")
arrows.circular(east_goal, col = "orange")
arrows.circular(west_goal, col = "orange")

# check the behavior assigned to this movement

df$behavior[1]

>> "drift"
```

References

1. Pearse AT, Rabbe M, Juliusson LM, Bidwell MT, Craig-Moore L, Brandt DA, et al. Delineating and identifying long-term changes in the whooping crane (Grus americana) migration corridor. PLOS ONE. Public Library of Science; 2018;13:e0192737.

2. Safi K, Kranstauber B, Weinzierl R, Griffin L, Rees EC, Cabot D, et al. Flying with the wind: scale dependency of speed and direction measurements in modelling wind support in avian flight. Mov Ecol. 2013;1:4.

3. Hesselbarth MHK, Sciaini M, With KA, Wiegand K, Nowosad J. landscapemetrics: an open-source R tool to calculate landscape metrics. Ecography. 2019;42:1648–57.

4. Douglas D, Weinzierl R, Davidson S, Kays R, Wikelski M, Bohrer G. Moderating Argos location errors in animal tracking data. Methods in Ecology and Evolution. 2012;3:999–1007.

Results

Supplementary Table 1: **LMMs evaluating the effect of wind support on measured ground speeds**

Model (fixed effect)	logLik	AIC_c	ΔAIC_c	weight	σ_{LMM}	$R^2_{LMM(f)}$
Max wind support (5-level, pdm)	-1904.009	3816.1	0.00	0.991	3.800	0.371
Max wind support (7-level, pdm)	-1908.721	3825.5	9.42	0.009	3.831	0.363
Max wind support (5-level, rt)	-1914.612	3837.3	21.21	0.000	3.858	0.350
Max wind support (7-level, rt)	-1930.645	3869.3	53.27	0.000	3.948	0.319
Fixed-altitude wind support (750, pdm)	-1930.795	3869.6	53.57	0.000	3.953	0.320
Fixed-altitude wind support (100, pdm)	-1947.917	3903.9	87.82	0.000	4.042	0.281
Fixed-altitude wind support (1500, pdm)	-1972.734	3953.5	137.45	0.000	4.188	0.269

Fixed effect reflects one of seven possible altitude-selection strategies, including fixed-altitude flight (at 100, 750, or 1500 m.a.s.l.) or selection of the altitude with maximum wind support (among 100, 750, 1500, 2250, 3000 m.a.s.l. for 5-level models, and additionally 3400 and 5500 m.a.s.l. for 7-level models), as measured along the preferred direction of movement (pdm) or along the realized track (rt). All models fit by maximum likelihood with a random intercept of individual.

Supplementary Table 2: Model estimates for angular difference between realized and preferred directions in godwit northward migration

Model term	Estimate	GCV	RSS	GRSq	R^2_{MARS}
(Intercept)	0.1415				
h(lat- -9.21)	0.009853				
h(cw- -0.43)	0.02991				
h(4.32-lat) * h(cw- -0.43)	-0.0009388				
h(lat-4.32) * h(cw- -0.43)	-0.001162				
h(lat- -9.21) * h(ws- -0.031)	-0.003434				
h(lat- -9.21) * h(-0.031-ws)	-0.001801				
h(lat- -9.21) * h(ws-1.91)	0.003246				
		0.0309	20.14	0.110	0.154

Results of multivariate adaptive regression spline (MARS) model selection procedure. Selected dependent variables are latitude (lat), crosswind (cw), and wind support (ws), with changepoints (h) shown above. GCV is generalized cross-validation; GRSq is an estimation of the predictive power of the model.

Supplementary Table 3: Model coefficients as estimated by LMMs for godwit forward and lateral movement

Model	Model term	Estimate	95% CI	t value	p	$R^2_{LMM(f)}$
$r_v \sim ws$	(Intercept)	12.061	11.479, 12.418	24.54		
	βws	0.799	0.739, 0.895	20.24	< 0.001	
	σ_{LMM}	3.80	3.50, 3.92			
						**0.383**
$r_u \sim cw$	(Intercept)	-0.330	-0.738, 0.077	-1.583		
	βcw	0.315	0.240, 0.384	8.321		
	σ_{LMM}	3.71	3.51, 3.91		< 0.001	
						**0.096**

Godwit forward movement in the preferred direction (r_v, ms^{-1}) as predicted by wind support in the (ws, ms^{-1}). Godwit lateral movement in the preferred direction (r_u, ms^{-1}) as predicted by crosswinds (cw, ms^{-1}). Model fit by maximum likelihood with a random intercept of individual ID and year ($r_v \sim ws$) or individual ID ($r_v \sim ws$). R^2 shown for fixed effects only.

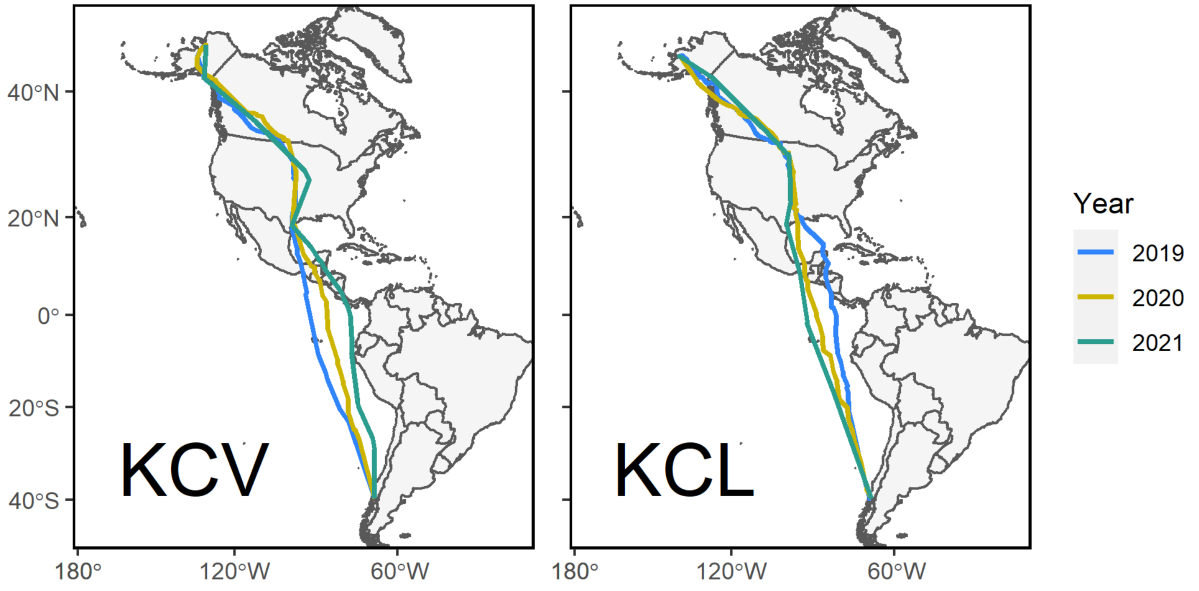

Supplementary Figure 1 Movement tracks recorded for two individuals ("KCL" and "KCV") over three years of northward migratory flight.

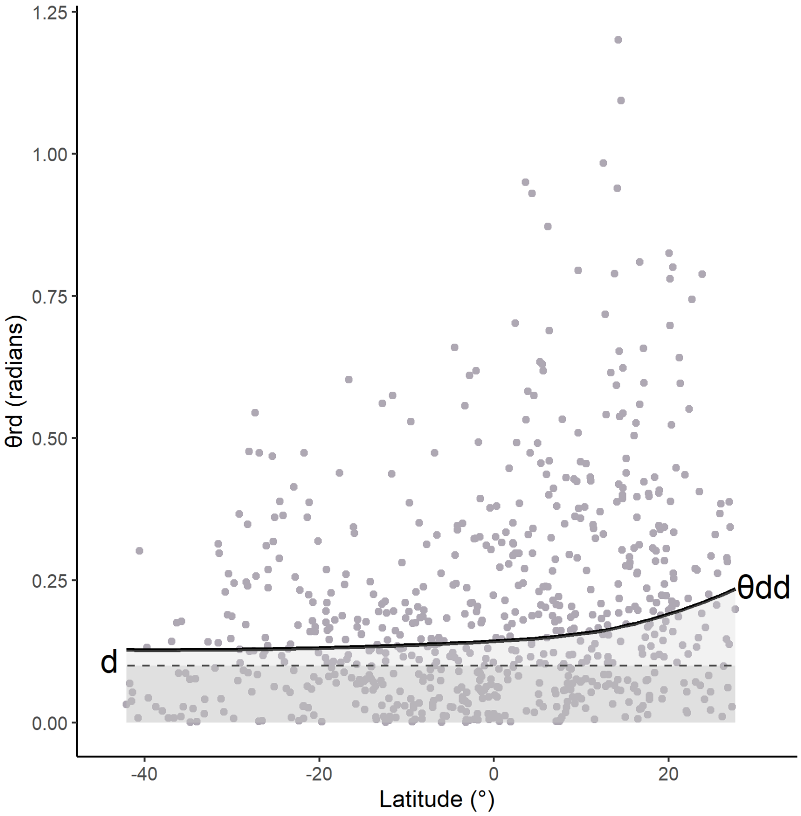

Supplementary Figure 2 Effect of classifying full compensation using preferred direction versus range of the preferred direction in movements of tracked godwits. Points indicate observations of angular difference between realized and preferred directions (θ_{rd}) at given latitudes. Dashed horizontal line (d) indicates the cut-off point for full compensation in a conventional, fixed destination model; all points below this line would be considered full compensation. Solid line (θ_{dd}) indicates the cut-off point for full compensation in our circular model, which utilizes a range of the preferred direction that increases with proximity.

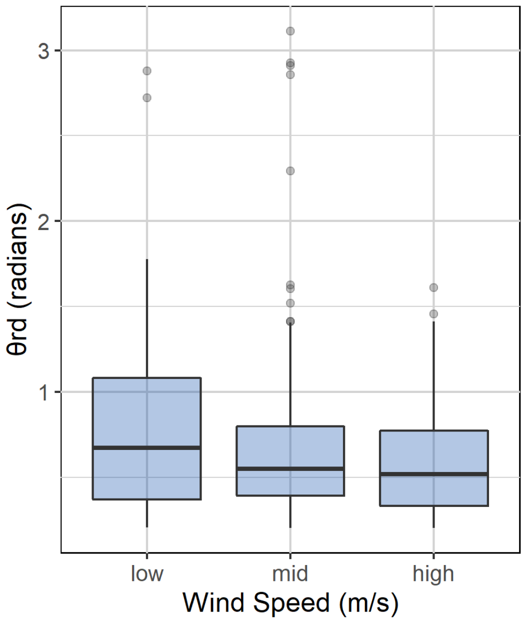

Supplementary Figure 3 For movements classified as *fully compensating* (n=283), godwit divergence from the preferred direction (θ_{rd}) at varying wind speeds. Wind speeds are considered low (speed<3.76 ms^{-1}, equal to 25th percentile; 23.0%), mid (3.76 ms^{-1}<speed<8.29 ms^{-1}, equal to IQR; 54.8%), or high (speed>8.29 ms^{-1}, equal to 75th percentile; 22.2%).
